# Vitamin D Receptor Polymorphism and Myasthenia Gravis in Chinese Han Population

**DOI:** 10.3389/fneur.2021.604052

**Published:** 2021-02-09

**Authors:** Ji-Lan Han, Yao-Xian Yue, Xiang Gao, Yan-Chen Xie, Hong-Jun Hao, Hong-Yan Li, Xiao-Long Yu, Jie Li, Rui-Sheng Duan, Hai-Feng Li

**Affiliations:** ^1^Department of Neurology, Shandong Provincial Qianfoshan Hospital, Cheeloo College of Medicine, Shandong University, Jinan, China; ^2^Department of Neurology, Weifang People's Hospital, Weifang, China; ^3^Department of Neurology, Qilu Hospital, Cheeloo College of Medicine, Shandong University, Jinan, China; ^4^Department of Allergy, Affiliated Hospital of Qingdao University, Qingdao, China; ^5^Department of Neurology, Beijing Friendship Hospital, Capital Medical University, Beijing, China; ^6^Department of Neurology, Peking University First Hospital, Beijing, China; ^7^Department of Neurology, Affiliated Qingdao Central Hospital, Qingdao University, Qingdao, China; ^8^Department of Neurology, Xuanwu Hospital, Capital Medical University, Beijing, China

**Keywords:** myasthenia gravis, vitamin D receptor, polymorphism, susceptibility, severity

## Abstract

Myasthenia gravis (MG) is an autoimmune disease in which antibodies bind to acetylcholine receptors (AChR) or other functional molecules in the postsynaptic membrane at the neuromuscular junction. Vitamin D (VD) has a number of pluripotent effects, which include immune-regulation and bone metabolism. The immunomodulatory actions of 1,25(OH)2D3 are mediated by its binding to a vitamin D receptor (VDR). In the study, we undertook a case-control study to explore the association between VDR gene polymorphism and the susceptibility and severity of MG patients. Four hundred and eighty MG patients and 487 healthy controls were included and gene polymorphisms of VDR were determined with improved multiplex ligation detection reaction technique and SNPscan^TM^ technique. MG patients were classified into subgroups by essential clinical features and by a comprehensive classification. The frequencies of alleles and genotypes were compared between the MG group and the control group, between each MG subgroup and the control group, and between each pair of MG subgroups. There were no significant differences in frequencies of alleles and genotypes between MG patients and healthy controls, between MG subgroups and healthy controls, or between each pair of MG subgroups in the analysis of subgroups classified by essential clinical features (onset age, gender, thymoma, AChRAb positivity, onset involvement) and the maximal severity (modified Oosterhuis score). In the analysis of subgroups with a comprehensive classification, the frequencies of alleles and genotypes in rs731236 showed significant differences between adult non-thymoma AChRAb negative MG subgroup and the control group, as well as the adult non-thymoma AChRAb positive MG group. In the Chinese Han population, rs731236 was found to be possibly associated with adult non-thymoma AChRAb negative MG patients, although this needs further confirmation.

## Introduction

Myasthenia gravis (MG) is an autoimmune disease in which antibodies bind to acetylcholine receptors (AChR) or to other functional molecules, such as muscle-specific kinase (MuSK) and lipoprotein-receptor-related protein 4 (LRP4), in the postsynaptic membrane at the neuromuscular junction (NMJ) (1). Various immune cells involving innate and adaptive immunity participate in the pathogenesis of MG, including dendritic cells and B and T lymphocytes (2). Immune-modulating molecules, such as cytokines, are major mediators. An aberrant regulation of the immune system is presumed to be involved in the susceptibility and the severity of MG. Immune response is also modulated by other molecules, such as vitamin D (3).

Vitamin D (VD) has a number of pluripotent effects, which include immune-regulation and bone metabolism. 1,25(OH)2D3 promotes the differentiation of monocytes and inhibits the maturation of dendritic cells (4). 1,25(OH)2D3 inhibits the proliferation and differentiation of T helper 1 cells and modulates cytokine production by reducing the expression of pro-inflammatory interleukin-2 and interferon-γ, stimulates T helper 2 cells with upregulation of the production of anti-inflammatory cytokines, suppresses the development of Th17 cells, inhibits the production of interleukin-17, and induces proliferation of regulatory T cells (5, 6). Moreover, 1,25(OH)2D3 inhibits proliferation and differentiation of plasma cells (6).

The immunomodulatory actions of 1,25(OH)2D3 are mediated by its binding to VDR. The Vitamin D receptor (VDR) is a transcription factor belonging to the glucocorticoid nuclear receptor family, which binds to 1α,25-dihydroxyvitamin D3 [1,25(OH)2D3] (7). VDRs are expressed in most immune cells, including monocytes/macrophages, dendritic cells, B lymphocytes, and T lymphocytes (3). The formation of the VD-VDR complex results in gene expression at the transcriptional level (3). The VDR gene is located in 12q14, which includes six untranslated exons (exon1a-1f) and eight coding exons (exons 2–9). The VDR gene could affect the expression of VDR (8). VDR genes' polymorphism has been associated with many autoimmune disorders such as autoimmune thyroid disease (9), idiopathic inflammatory myopathy (10), multiple sclerosis (11), type 1 diabetes mellitus (12), and systemic lupus erythematosus (13).

Our previous study found that VDR gene Tru9I (rs757343) polymorphism was associated with risk of MG in females older than 15 years (14). In this study, we undertook a case-control study to further explore the association between VDR gene polymorphisms and the susceptibility and severity of MG patients in a systematic way.

## Subjects and Methods

### Subjects

Four hundred and eighty patients who were diagnosed and treated in the Affiliated Hospital of Qingdao University and Beijing Friendship Hospital, Capital Medical University and 487 randomly recruited healthy controls in the same area were included in this study. All MG patients met the following diagnostic criteria: typical symptoms of fluctuating muscle weakness, positive result of neostigmine test, and the presence of AChRAb or MuSK antibody and/or amplitude decrement response >10% in low frequency repetitive nerve stimulation test (15, 16). MG patients were followed up with at least twice a year, with additional follow-ups if symptoms worsened and within 2–3 months thereafter (15, 16). Maximal severity was acquired by the history of MG patients and follow-ups and qualified by Oosterhuis score (17). Chronic infections (with relevant tests to exclude suspected infection when possible) and commonly associated autoimmune diseases (by self-reported questionnaire and tests including anti-thyroid antibodies and ENA) were excluded in both MG patients and healthy controls. All MG patients and healthy controls were Han Chinese in origin and non-consanguineous. Informed consent was obtained from all adult participants and the guardians of juvenile MG patients. The study was approved by the ethical committees of the two hospitals.

**Figure 1:**
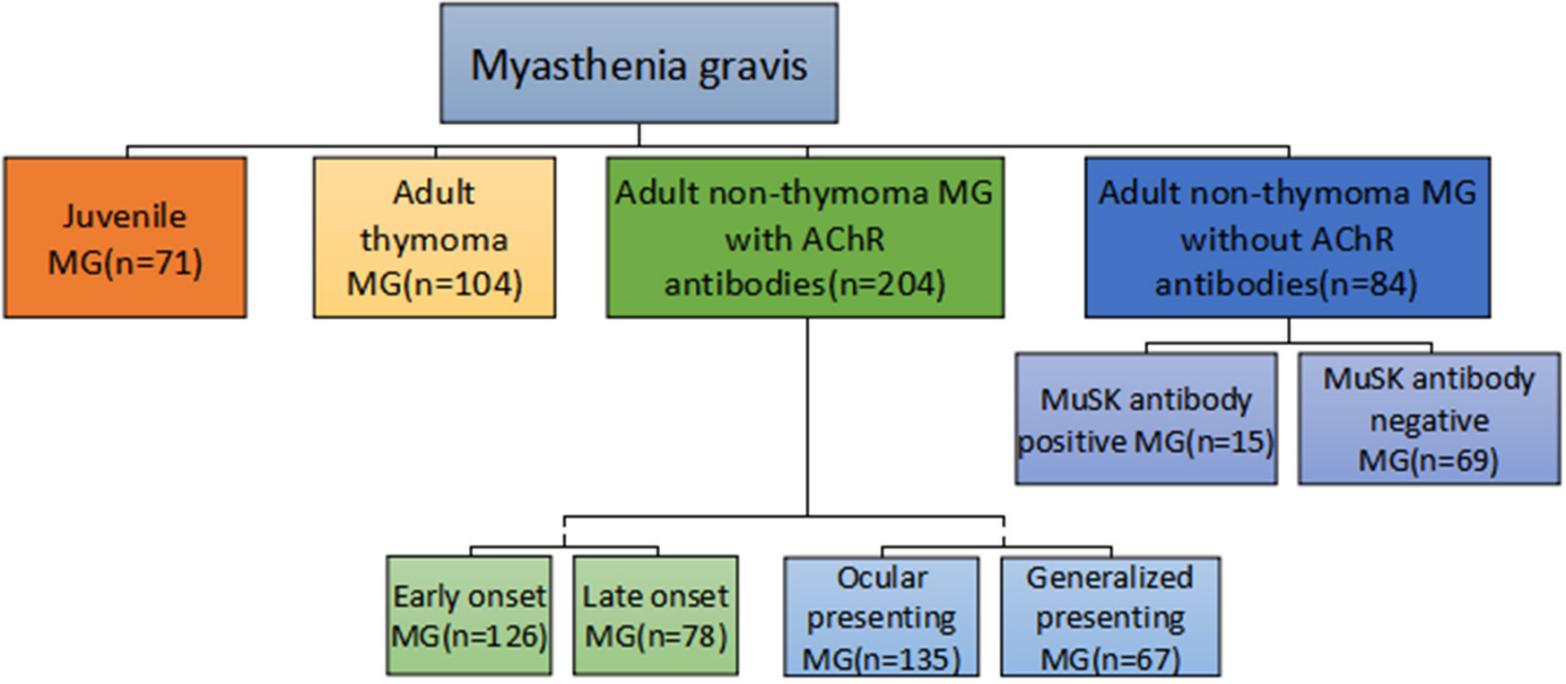
MG comprehensive subgroup classification.

Patients were classified by essential clinical characteristics such as gender, onset age (<15/15–50/>50 years) (1), thymoma (typical CT and/or pathology), AChRAb, onset involvement (ocular/generalized), and the maximal severity (modified Oosterhuis score 0–2/3–5). AChRAb was detected with ELISA kits (RSR Limited, Cardiff, UK) and MuSK antibodies were detected and measured with RIA method (RSR Limited, Cardiff, UK) in AChR antibody negative MG patients. A natural history study showed that 82% of MG patients reached maximum worsening within 2 years after onset (18); therefore, the maximum Oosterhuis score was analyzed only in patients with a clinical course of 2 years or more. Because of potential interactions among the essential clinical characteristics (16, 19), a comprehensive classification (Figure 1) (20) was also used in the analysis. The new classification used the combination of essential clinical characteristics of MG to classify biologically and clinically meaningful subgroups. It ensured that any MG patients with sufficient data were assigned into one subgroup and only to one subgroup.

### Methods

#### SNP Selection and Genotyping

SNPs were selected systematically, including the functional loci [rs4516035 (5′ near gene), rs2228570 (exon 2), rs9729 (3′UTR)], hot SNPs [rs1544410 (9, 10, 12), rs731236 (9, 10, 12), rs7975232 (9, 10, 12), rs757343 (21), rs2238136 (22)], and tag SNPs (rs3847987, rs10875692, rs2107301, rs2239186, rs2853564, rs11574027, rs7136534, rs739837, and rs2239181). Nine tag SNPs were selected using the UCLA Association Study Design Server online software package (http://design.cs.ucla.edu), based on HapMap database (23). The minor allele frequency (MAF) of each SNP was more than 5% in the Han Chinese population (1,000 Genomes Project Phase 3). Genomic DNA was extracted using peripheral blood genomic DNA purification kit (Biochain, Newark, CA, USA). Rs1544410 and rs2107301 were genotyped by using improved multiplex ligation detection reaction (iMLDR) technique (Shanghai Genesky Biotechnologies Inc. China). The remaining fifteen SNPs were genotyped with SNPscan^TM^ technique Kit (Cat#: G0104, Genesky Biotechnologies Inc. Shanghai, China). The primers and probes will be provided on request. Forty samples were randomly selected for double-blind quality control, and the results were consistent with the original genotyping results.

### Statistical Analysis

The online SNPstats software (https://snpstats.net/start.htm) was used to test the Hardy-Weinberg equilibrium in the control group. Genotype frequencies were analyzed under codominant and additive inheritance models in SNPstats software. The χ^2^ test or Fisher exact test was used to compare the allele frequencies between MG group and the control group, between each MG subgroup and the control group, and between each pair of MG subgroups (SPSS17.0). Bonferroni correction was applied for the multiple-testing. When there were significant differences in allele frequencies between MG subgroups and the control group or among MG subgroups, Logistic regression (SPSS 17.0) was used to adjust for potential confounding factors. The Haploview 4.2 software was used to calculate the linkage disequilibrium of SNPs and construct haplotype blocks. *Post-hoc* statistical power was calculated by Quanto program (version 1.2.4). *P* ≤ 0.05 was considered as statistically significant.

## Results

### General Characteristics

The successful genotyping rates of the seventeen SNPs were 96.07%~99.79%. Among the 17 selected SNPs, frequencies of rs2853564 in the healthy controls (*P* < 0.05) were not consistent with the Hardy-Weinberg equilibrium and were excluded from further analysis (Table 1).

**Table 1:** General information of SNPs in MG patients and healthy controls.

**SNPs**	**Function**	**Alleles**	**Allele frequencies**		***P-*value**	**Genotypes**	**Genotype frequencies**		***P-*value**	
**(Genotyping rate)**			**MG**	**HC**			**MG**	**HC**	**Codominant**	**Additive**
rs4516035	5′near gene	T	929 (0.98)	939 (0.97)	0.280	TT	454 (0.96)	456 (0.94)	0.36	0.28
(99.17%)		C	21 (0.02)	29 (0.03)		TC	21 (0.04)	27 (0.06)		
						CC	0 (0)	1 (0)		
rs7136534	Intron	C	574 (0.61)	601 (0.62)	0.564	CC	178 (0.38)	184 (0.38)	0.61	0.57
(99.28%)		T	374 (0.39)	371 (0.38)		CT	218 (0.46)	233 (0.48)		
						TT	78 (0.16)	69 (0.14)		
rs11574027	Intron	C	829 (0.87)	852 (0.88)	0.796	AA	4 (0.01)	9 (0.02)	0.25	0.79
(99.38%)		A	121 (0.13)	120 (0.12)		CA	113 (0.24)	102 (0.21)		
						CC	358 (0.75)	375 (0.77)		
rs2238136	Intron	C	763 (0.8)	775 (0.8)	0.682	CC	304 (0.64)	314 (0.64)	0.27	0.69
(99.48%)		T	187 (0.2)	199 (0.2)		CT	155 (0.33)	147 (0.3)		
						TT	16 (0.03)	26 (0.05)		
rs2228570	Missense	G	531 (0.56)	529 (0.54)	0.422	AA	79 (0.17)	102 (0.21)	0.18	0.41
(99.07%)	Mutation	A	413 (0.44)	443 (0.46)		GA	255 (0.54)	239 (0.49)		
						GG	138 (0.29)	145 (0.3)		
rs2239186	Intron	A	484 (0.51)	490 (0.5)	0.637	AA	126 (0.27)	122 (0.25)	0.83	0.64
(98.86%)		G	456 (0.49)	482 (0.5)		AG	232 (0.49)	246 (0.51)		
						GG	112 (0.24)	118 (0.24)		
rs2239181	Intron	A	742 (0.78)	789 (0.81)	0.161	AA	289 (0.61)	324 (0.67)	0.16	0.16
(99.28%)		C	204 (0.22)	185 (0.19)		AC	164 (0.35)	141 (0.29)		
						CC	20 (0.04)	22 (0.05)		
rs2107301	Intron	A	665 (0.7)	681 (0.7)	0.864	AA	236 (0.49)	233 (0.48)	0.32	0.86
(99.79%)		G	291 (0.3)	293 (0.3)		AG	193 (0.4)	215 (0.44)		
						GG	49 (0.1)	39 (0.08)		
rs1544410	Intron	C	899 (0.94)	932 (0.96)	0.144	CC	422 (0.88)	445 (0.91)	0.13	
(99.69%)		T	55 (0.06)	42 (0.04)		CT	55 (0.12)	42 (0.09)		
rs757343	Intron	C	717 (0.78)	749 (0.79)	0.632	CC	278 (0.6)	295 (0.62)	0.87	0.63
(97.1%)		T	207 (0.22)	205 (0.21)		CT	161 (0.35)	159 (0.33)		
						TT	23 (0.05)	23 (0.05)		
rs10875692	Intron	C	796 (0.84)	798 (0.82)	0.279	CC	333 (0.7)	329 (0.68)	0.48	0.28
(99.48%)		T	154 (0.16)	176 (0.18)		CT	130 (0.27)	140 (0.29)		
						TT	12 (0.03)	18 (0.04)		
rs7975232	Intron	C	650 (0.71)	713 (0.74)	0.218	AA	36 (0.08)	39 (0.08)	0.22	0.22
(97.31%)		A	264 (0.29)	255 (0.26)		CA	192 (0.42)	177 (0.37)		
						CC	229 (0.5)	268 (0.55)		
rs731236	Synonymous	A	895 (0.94)	928 (0.95)	0.294	AA	422 (0.89)	442 (0.91)	0.56	0.3
(99.48%)	Mutation	G	55 (0.06)	46 (0.05)		AG	51 (0.11)	44 (0.09)		
						GG	2 (0)	1 (0)		
rs739837	3′UTR	G	640 (0.71)	704 (0.73)	0.253	GG	226 (0.5)	265 (0.55)	0.23	0.26
(96.07%)		T	260 (0.29)	254 (0.27)		GT	188 (0.42)	174 (0.36)		
						TT	36 (0.08)	40 (0.08)		
rs3847987	3′UTR	C	732 (0.77)	763 (0.78)	0.499	AA	24 (0.05)	24 (0.05)	0.73	0.5
(99.48%)		A	218 (0.23)	211 (0.22)		CA	170 (0.36)	163 (0.33)		
						CC	281 (0.59)	300 (0.62)		
rs9729	3′UTR	G	666 (0.7)	704 (0.73)	0.180	GG	235 (0.49)	256 (0.53)	0.4	0.18
(99.48%)		T	288 (0.3)	266 (0.27)		GT	196 (0.41)	192 (0.4)		
						TT	46 (0.1)	37 (0.08)		

In MG patients, 189 were males and 291 were females. Onset age was 1–86 years old (median 40, interquartile range 32). The disease duration of MG ranged from 8 to 220 months (median 43, interquartile range 61). There were 107 patients with thymoma and 367 patients without thymoma. Three hundred and thirty eight patients were AChRAb positive and 124 patients were AChRAb negative. Three hundred and forty two patients were ocular presenting and 135 patients were generalized presenting at onset. The others had no relevant information. The maximum Oosterhuis score was available in 370 patients (77%). Two hundred and sixteen patients were classified into the mild subgroup (Oosterhuis score 0–2) and 154 patients into the severe subgroup (Oosterhuis score 3–5) (Supplementary Table 1).

Four hundred and eighty seven healthy controls, including 249 males and 238 females, were 14–78 years old (median 45, interquartile range 24).

### Allele and Genotype Frequency Comparison in MG Group/Each MG Subgroup and the Control Group

There were no significant differences in allele frequencies between the MG group and the control group, and in genotype frequencies between the MG group and the control group under the codominant or additive inheritance model among the 16 SNPs. There were no significant differences in allele or genotype frequencies between each MG subgroup (gender, onset age, thymoma, AChRAb, onset involvement, and maximal severity) and the control group, or between each pair of MG subgroups (Supplementary Table 1).

### Allele and Genotype Frequency Comparison in the Comprehensive Classified MG Subgroups and the Control Group

According to the comprehensive classification, 71 patients were juvenile MG (onset age <15) and 409 patients were adult MG. In adult MG patients, 104 patients had thymoma and 300 patients were without thymoma. In adult non-thymoma patients, 84 patients were AChRAb negative and 204 patients were AChRAb positive. In adult non-thymoma AChRAb negative patients, 15 patients were MuSK antibody positive and 69 patients were MuSK antibody negative. In adult non-thymoma AChRAb positive patients, 126 patients were early onset MG (EOMG, onset age 15–50) and 78 patients were late onset MG (LOMG, onset age >50); 135 patients were ocular presenting and 67 patients were generalized presenting. The others had no relevant information (Supplementary Table 2).

The G allele frequency in rs731236 was significantly higher in the adult non-thymoma AChRAb negative MG subgroup than that in the control group (P_bon_ = 0.032, OR = 2.42) and in the adult non-thymoma AChRAb positive MG group (P_bon_ = 0.032, OR = 2.90) (Table 2). *Post-hoc* statistical power was 0.9791 and 0.9929 based on Log-additive inheritance mode for the two comparisons. There were significant differences in genotype frequencies between adult non-thymoma AChRAb negative MG and the control group (*P* = 0.017 and *P* = 0.0044) as well as between adult non-thymoma AChRAb negative MG and adult non-thymoma AChRAb positive MG (*P* = 0.0092 and *P* = 0.003) in rs731236 under the codominant and additive inheritance model (Table 2).

**Table 2:** Significant differences in subgroups and healthy controls.

	**Number^**a**^**	***P-*value^**b**^**	**P_**bon**_**	**OR**	**95%CI**	**Codominant^**c**^**	**Additive**
rs731236							
Adult thymoma (–) AChRAb (–) MG/control group	84/487	0.002	0.032	2.42	1.37–3.75	0.017	0.0044
Adult thymoma (–) AChRAb (–) MG/adult thymoma (–) AChRAb (+) MG	84/201	0.002	0.032	2.9	1.44–4.82	0.0092	0.003

a *successfully genotyped number with adequate clinical data in each group*.

b *P-value, P_bon_, the OR, and 95%CI in allele frequency comparison between MG subgroup and the control group*.

c *P-value in genotype frequency comparison under codominant and additive inheritance models*.

We further compared between the MuSK antibody positive and MuSK antibody negative (double negative) group within the AChRAb negative MG patients. The G allele frequency in rs731236 was significantly higher in the double negative group than those in the control group (P_bon_ = 0.016, OR = 2.65). There were no significant differences in allele frequency in rs731236 between the MuSK positive and the double negative group, as well as between the MuSK positive and the control group (Supplementary Table 2).

### Adjustment of Potential Confounding Factors in Clinical Variable Based Subgroup Analysis

Logistic regression analysis was performed in adult non-thymoma MG patients with AChRAb (positive and negative) as a dependent variable, and with gender (male and female), onset age (15–50 and >50 years), muscle involvement at onset (ocular and generalized), and genotypes of rs731236 (codominant model) as independent variables. The genotype and muscle involvement at onset were found to be independent risk factors (Table 3).

**Table 3:** Logistic regression analysis in subgroups.

**Variables**	**Regression coefficient**	**Standard error**	**Wald x^**2**^ value**	***P*-value**	**OR**
Onset age	0.261	0.272	0.924	0.337	1.298
Gender	−0.322	0.275	1.366	0.243	0.725
Muscle involvement at onset	−0.629	0.320	3.857	0.050	0.533
Rs731236	1.046	0.372	7.914	0.005	2.847

### Linkage Disequilibrium Analysis

**Figure 2:**
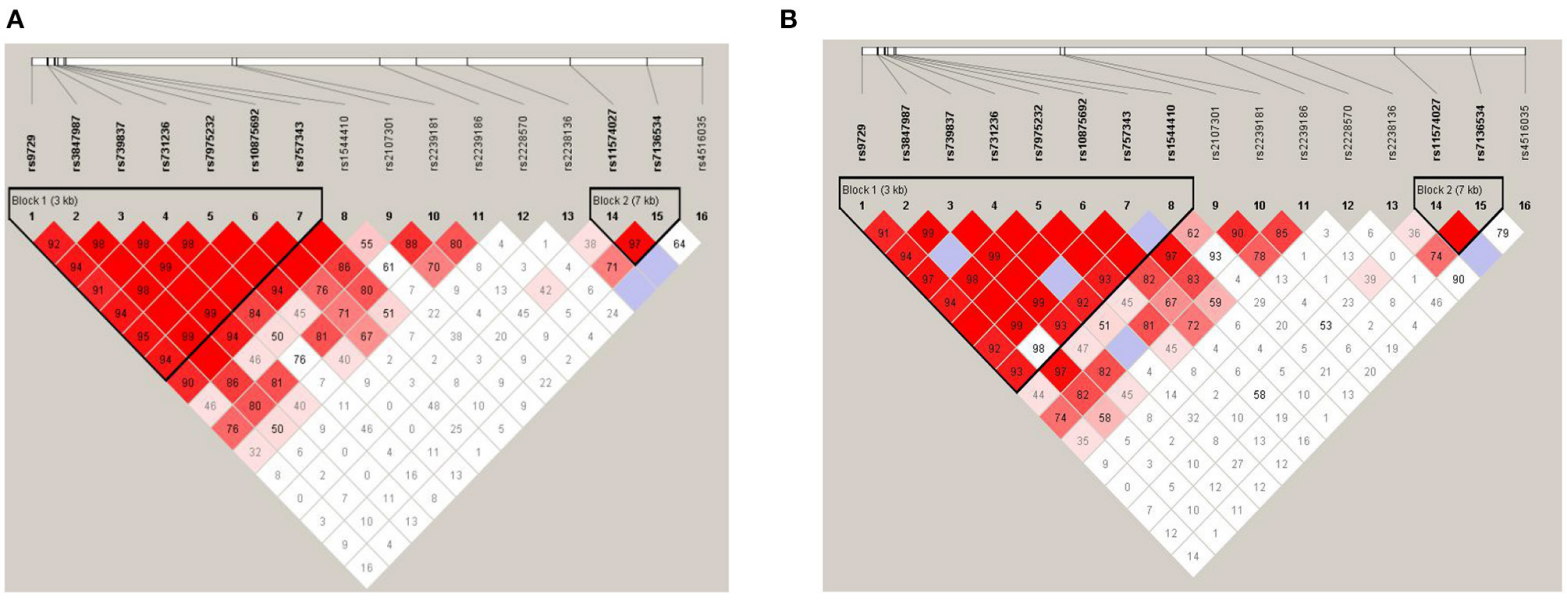
Linkage disequilibrium (LD) plot and haplotype block construction. MG patients and healthy controls **(A)**; adult non-thymoma AChRAb negative MG patients and healthy controls **(B)**. The locations of each SNP were indicated by the straight lines. The D' value corresponding to each SNP pair was expressed as a percentage and shown within the respective square (D' = 1, not shown). Higher D' values were indicated in darker red.

Linkage disequilibrium analysis amongst 16 SNPs was shown in Figure 2A. Two haplotype blocks were constructed. Block 1 was constructed by rs9729, rs3847987, rs739837, rs731236, rs7975232, rs10875692, and rs757343, and block 2 was constructed by rs11574027 and rs7136534. There were no significant differences in haplotype frequencies between MG and the control group (Table 4.1). We also performed linkage disequilibrium analysis between the adult non-thymoma AChRAb negative MG subgroup and the control group (Figure 2B). Two haplotype blocks were constructed. Block 1 was constructed by rs9729, rs3847987, rs739837, rs731236, rs7975232, rs10875692, rs757343, and rs1544410, and block 2 was constructed by rs11574027 and rs7136534. There were also no significant differences in haplotype frequencies between the adult non-thymoma AChRAb negative MG subgroup and the control group (Table 4.2).

**Table 4.1:** Haplotypes of the VDR gene between MG and the control group.

**Haplotypes^**a**^**	**MG freq**.	**Control freq**.	**Chi square**	***P-*value**
Block 1				
GCGACCC	0.526	0.540	0.359	0.549
TATAACT	0.214	0.208	0.132	0.7161
GCGACTC	0.159	0.179	1.287	0.2566
TCTGACC	0.053	0.047	0.308	0.5789
TCGACCC	0.014	0.016	0.106	0.7452
Block 2				
CC	0.480	0.496	0.473	0.4917
CT	0.392	0.381	0.279	0.5976
AC	0.126	0.123	0.04	0.8417

a *haplotypes with frequency <1% were not listed*.

*The SNP sequences are rs9729-rs3847987-rs739837-rs731236-rs7975232-rs10875692- rs757343 in block 1 and rs11574027-rs7136534 in block 2*.

**Table 4.2:** Haplotypes of the VDR gene between adult non-thymoma AChRAb negative MG and the control group.

**Haplotypes^**a**^**	**MG freq**.	**Control freq**.	**Chi square**	***P-*value**
Block 1				
GTGATTTT	0.532	0.538	0.021	0.8846
CACAATCT	0.173	0.208	1.041	0.3076
GTGATCTT	0.147	0.182	1.073	0.3003
CTCGATTC	0.077	0.043	3.523	0.0605
CTGATTTT	0.019	0.018	0.017	0.8957
Block 2				
TT	0.476	0.495	0.212	0.6452
TC	0.373	0.381	0.037	0.8472
AT	0.151	0.123	0.943	0.3315

a *haplotypes with frequency <1% were not listed*.

*The SNP sequences are rs9729-rs3847987-rs739837-rs731236-rs7975232-rs10875692-rs757343-rs1544410 in block 1 and rs11574027-rs7136534 in block 2*.

## Discussion

In our study, we found there were no significant differences between MG patients and healthy controls, between MG subgroups and healthy controls, or between each pair of MG subgroups in the analysis of subgroups classified by essential clinical features and the maximal severity. In subgroup analysis by the comprehensive classification, the frequencies of alleles and genotypes in rs731236 showed significant differences between the adult non-thymoma AChRAb negative MG subgroup and the control group, as well as the adult non-thymoma AChRAb positive MG group (Table 2). The statistical power of this association was high. We further compared between the MuSK antibody positive and the double negative group within the AChRAb antibody negative MG patients. There was significant difference between the double negative group and the control group. However, there were no significant differences between the MuSK antibody positive group and double negative group, as well as the control group. Although the comprehensive classification eliminates some of the confounding factors, other clinical variables (early or late onset, gender, and initial muscular involvement) might lead to confounding effects. Logistic analysis revealed that rs731236 was an independent risk factor in the adult non-thymoma AChRAb negative MG subgroup.

Rs731236 (also known as TaqI) is within exon 9. Its allele polymorphism yields a synonymous coding sequence. Nevertheless, it is located near the 3′UTR of VDR, which is known to be involved in the regulation of gene expression through the regulation of mRNA stability and protein translation efficiency (8). The rs731236 was found to be located in a CpG imposing a direct *cis* effect on site-specific and regional methylation (24). Children carrying the C allele for TaqI were more likely to develop asthma, and interleukin-10 levels were significantly low in asthmatics with the TC genotype for TaqI due to a decrease in expression of VDR (25). Therefore, it is presumed that rs731236 can affect the expression of VDR, and thus affect the expression of cytokines, thereby exerting immunomodulatory effects. rs731236 was also found to be associated with allergic diseases (8), autoimmune thyroid disease (9), and multiple sclerosis (26).

MG is a heterogeneous autoimmune disease with distinct immunogenetic characteristics in different MG subtypes (27). In AChRAb negative MG patients, antibodies against other NMJ proteins, such as MuSK and LRP4, are found (28). Moreover, some of the AChR antibody negative patients might be shown to be AChR antibody positive when more sensitive testing methods are used (29). In our study, there were no significant differences between the MuSK antibody positive and double negative group, as well as the control group. Hence, the double (AChR and MuSK antibodies) negative MG patients should be analyzed with further antibody testing.

Previous studies found that rs7975232, rs731236, and rs1544410 variants were in strong linkage disequilibrium (30), and we also performed haplotype analysis. We found that rs9729, rs3847987, rs739837, rs731236, rs7975232, rs10875692, and rs757343 were in high linkage disequilibrium, but there were no significant differences in haplotype frequencies between MG and the control group, which suggested that these haplotypes were not significantly related to the susceptibility of MG. There were also no significant differences in haplotype frequencies between the adult non-thymoma AChRAb negative MG subgroup and the control group.

Our previous research (14) found that VDR gene Tru9I (rs757343) polymorphism may be associated with risk of MG in females older than 15 years. However, the association was only found in a subgroup and the sample size was small. Moreover, AChR antibodies were not included in that study and no Logistic analysis was performed, which might lead to bias. Therefore, we recruited a new cohort with a larger sample size, selected SNPs of VDR gene in a systematic strategy, and performed Logistic analysis. In this study, we used both the subgroups classified by single clinical features and a new comprehensive scheme. We cannot confirm the previous association in this study.

There are several limitations to our study. The first limitation is that we did not measure the serum vitamin D levels. Our study mainly explores the association between vitamin D receptor gene polymorphism and the susceptibility and maximal severity of MG. Although vitamin D levels may be related with severity of MG by its immune-modulating effects, the serum vitamin D levels on blood collecting did not reflect the vitamin D levels during the entire disease course and at the maximal severity, due to factors such as steroid use, dietary habits, and sun exposure. This study cannot exclude the effects of vitamin D level by its design. Another limitation is that severity is analyzed by Oosterhuis score. It is relatively crude but has been used in other association studies (17). However, our study analyzes the maximum severity during the whole disease course of the patients. Oosterhuis scores can be accessed from the medical history, while more accurate measurements of severity, such as QMG scores recommended by MGFA, require on-site measurement, and hence are unfit for collecting information of maximal severity. A final limitation is that the sample size of the MuSK antibody positive MG subgroup is small and other related antibodies were not examined.

In conclusion, in the Chinese Han population, rs731236 was found to be possibly associated with adult non-thymoma AChRAb negative MG patients. Our study is a preliminary study, which needs further confirmation.

## Data Availability Statement

The datasets presented in this study can be found in online repositories. The names of the repository/repositories and accession number(s) can be found here: https://www.ncbi.nlm.nih.gov/SNP/snp_viewTable.cgi?handle=VDR-MG-CHINESE.

## Ethics Statement

The studies involving human participants were reviewed and approved by the ethical committees of Affiliated Hospital of Qingdao University, and Beijing Friendship Hospital, Capital Medical University. Written informed consent to participate in this study was provided by the participants' legal guardian/next of kin. Written informed consent was obtained from the individual(s), and minor(s)' legal guardian/next of kin, for the publication of any potentially identifiable images or data included in this article.

## Author Contributions

J-LH, Y-XY, and H-FL designed the study, interpreted the data, and wrote the manuscript. Y-XY and H-FL designed the experiment. J-LH and Y-XY performed statistical analysis. Y-CX and H-JH contributed to discussion. H-FL, Y-CX, and XG diagnosed and treated patients. XG, H-YL, X-LY, and JL maintained the database. R-SD and H-FL revised the manuscript. All authors approved the final manuscript.

## Conflict of Interest

The authors declare that the research was conducted in the absence of any commercial or financial relationships that could be construed as a potential conflict of interest.
