# Supplementary material for: Vitamin D Receptor Polymorphism and Myasthenia Gravis in Chinese Han Population
Source: Front Neurol. 2021 Feb 9;12:604052. doi: 10.3389/fneur.2021.604052 (PMC7900549; doi:10.3389/fneur.2021.604052)
Supplement: Supplementary file 1 [file Table_1.doc]

Table S1| Frequencies of alleles and genotypes in MG subgroups and the control group

|  | Control | Male | | Female | | Onset age (year) | | | Thymoma | | AChR-Ab | | Onset involvement | | Oosterhuis score | |
| --- | --- | --- | --- | --- | --- | --- | --- | --- | --- | --- | --- | --- | --- | --- | --- | --- |
|  |  | MG | control | MG | control | ＜15 | 15-50 | ＞50 | Positive | Negative | Positive | Negative | Ocular | Generalized | 0-2 | 3-5 |
| Number | 487 | 189 | 249 | 291 | 238 | 71 | 253 | 156 | 107 | 367 | 338 | 124 | 342 | 135 | 216 | 154 |
| rs4516035 | 484 | 185 | 247 | 290 | 237 | 71 | 252 | 152 | 106 | 363 | 334 | 123 | 338 | 134 | 216 | 152 |
| TT | 456(0.94) | 175(0.95) | 231(0.94) | 279(0.96) | 225(0.95) | 67(0.94) | 241(0.96) | 146(0.96) | 100(0.94) | 348(0.96) | 319(0.96) | 117(0.95) | 322(0.95) | 129(0.96) | 205(0.95) | 149(0.98) |
| TC | 27(0.06) | 10(0.05) | 16(0.06) | 11(0.04) | 11(0.05) | 4(0.06) | 11(0.04) | 6(0.04) | 6(0.06) | 15(0.04) | 15(0.04) | 6(0.05) | 16(0.05) | 5(0.04) | 11(0.05) | 3(0.02) |
| CC | 1(0) | 0 | 0 | 0 | 1(0) | 0 | 0 | 0 | 0 | 0 | 0 | 0 | 0 | 0 | 0 | 0 |
| C | 29(0.03) | 10(0.03) | 16(0.03) | 11(0.02) | 13(0.03) | 4(0.03) | 11(0.02) | 6(0.02) | 6(0.03) | 15(0.02) | 15(0.02) | 6(0.02) | 16(0.02) | 5(0.02) | 11(0.03) | 3(0.01) |
| rs7136534 | 486 | 185 | 248 | 289 | 238 | 69 | 252 | 153 | 106 | 362 | 333 | 124 | 337 | 134 | 216 | 152 |
| CC | 184(0.38) | 68(0.37) | 101(0.41) | 110(0.38) | 83(0.35) | 22(0.32) | 96(0.38) | 60(0.39) | 41(0.39) | 136(0.38) | 121(0.36) | 47(0.38) | 126(0.37) | 50(0.37) | 79(0.37) | 57(0.38) |
| CT | 233(0.48) | 85(0.46) | 112(0.45) | 133(0.46) | 121(0.51) | 36(0.52) | 111(0.44) | 71(0.46) | 47(0.44) | 169(0.47) | 157(0.47) | 56(0.45) | 159(0.47) | 59(0.44) | 98(0.45) | 70(0.46) |
| TT | 69(0.14) | 32(0.17) | 35(0.14) | 46(0.16) | 34(0.14) | 11(0.16) | 45(0.18) | 22(0.14) | 18(0.17) | 57(0.16) | 55(0.17) | 21(0.17) | 52(0.15) | 25(0.19) | 39(0.18) | 25(0.16) |
| T | 371(0.38) | 149(0.4) | 182(0.37) | 225(0.39) | 189(0.4) | 58(0.42) | 201(0.4) | 115(0.38) | 83(0.39) | 283(0.39) | 267(0.4) | 98(0.4) | 263(0.39) | 109(0.41) | 176(0.41) | 120(0.39) |
| rs11574027 | 486 | 185 | 248 | 290 | 238 | 70 | 252 | 152 | 106 | 363 | 334 | 123 | 338 | 134 | 216 | 152 |
| AA | 9(0.02) | 1(0.01) | 4(0.02) | 3(0.01) | 5(0.02) | 0 | 3(0.01) | 1(0.01) | 2(0.02) | 2(0.01) | 2(0.01) | 2(0.02) | 3(0.01) | 1(0.01) | 3(0.01) | 1(0.01) |
| CA | 102(0.21) | 44(0.24) | 65(0.26) | 69(0.24) | 37(0.16) | 13(0.18) | 60(0.24) | 40(0.26) | 25(0.24) | 86(0.24) | 80(0.24) | 30(0.24) | 79(0.23) | 33(0.25) | 49(0.23) | 32(0.21) |
| CC | 375(0.77) | 140(0.76) | 179(0.72) | 218(0.75) | 196(0.82) | 58(0.82) | 189(0.75) | 111(0.73) | 79(0.75) | 275(0.76) | 252(0.75) | 91(0.74) | 256(0.76) | 100(0.75) | 164(0.76) | 119(0.78) |
| A | 120(0.12) | 46(0.12) | 73(0.15) | 75(0.13) | 47(0.1) | 13(0.09) | 66(0.13) | 42(0.14) | 29(0.14) | 90(0.12) | 84(0.13) | 34(0.14) | 85(0.13) | 35(0.13) | 55(0.13) | 34(0.11) |
| rs2238136 | 487 | 185 | 249 | 290 | 238 | 71 | 252 | 152 | 106 | 363 | 334 | 123 | 338 | 134 | 216 | 152 |
| CC | 314(0.64) | 113(0.61) | 156(0.63) | 191(0.66) | 158(0.66) | 47(0.66) | 162(0.64) | 95(0.62) | 63(0.59) | 236(0.65) | 217(0.65) | 74(0.6) | 212(0.63) | 89(0.66) | 137(0.63) | 96(0.63) |
| CT | 147(0.3) | 68(0.37) | 78(0.31) | 87(0.3) | 69(0.29) | 21(0.3) | 84(0.33) | 50(0.33) | 37(0.35) | 117(0.32) | 104(0.31) | 46(0.37) | 114(0.34) | 41(0.31) | 73(0.34) | 51(0.34) |
| TT | 26(0.05) | 4(0.02) | 15(0.06) | 12(0.04) | 11(0.05) | 3(0.04) | 6(0.02) | 7(0.05) | 6(0.06) | 10(0.03) | 13(0.04) | 3(0.02) | 12(0.04) | 4(0.03) | 6(0.03) | 5(0.03) |
| T | 199(0.2) | 76(0.21) | 108(0.22) | 111(0.19) | 91(0.19) | 27(0.19) | 96(0.19) | 64(0.21) | 49(0.23) | 137(0.19) | 130(0.19) | 52(0.21) | 138(0.2) | 49(0.18) | 85(0.2) | 61(0.2) |
| rs2228570 | 486 | 184 | 249 | 288 | 237 | 71 | 250 | 151 | 105 | 361 | 331 | 123 | 336 | 133 | 214 | 152 |
| AA | 102(0.21) | 31(0.17) | 43(0.17) | 48(0.17) | 59(0.25) | 12(0.17) | 40(0.16) | 27(0.18) | 19(0.18) | 60(0.17) | 57(0.17) | 17(0.14) | 57(0.17) | 21(0.16) | 36(0.17) | 24(0.16) |
| GA | 239(0.49) | 103(0.56) | 121(0.49) | 152(0.53) | 118(0.5) | 35(0.49) | 137(0.55) | 83(0.55) | 58(0.55) | 193(0.53) | 169(0.51) | 78(0.63) | 183(0.54) | 71(0.53) | 110(0.51) | 87(0.57) |
| GG | 145(0.3) | 50(0.27) | 85(0.34) | 88(0.31) | 60(0.25) | 24(0.34) | 73(0.29) | 41(0.27) | 28(0.27) | 108(0.3) | 105(0.32) | 28(0.23) | 96(0.29) | 41(0.31) | 68(0.32) | 41(0.27) |
| A | 443(0.46) | 165(0.45) | 207(0.42) | 248(0.43) | 236(0.5) | 59(0.42) | 217(0.43) | 137(0.45) | 96(0.46) | 313(0.43) | 283(0.43) | 112(0.46) | 297(0.44) | 113(0.42) | 182(0.43) | 135(0.44) |
| rs2239186 | 486 | 183 | 248 | 287 | 238 | 70 | 249 | 151 | 105 | 359 | 331 | 121 | 335 | 132 | 212 | 151 |
| AA | 122(0.25) | 63(0.34) | 61(0.25) | 63(0.22) | 61(0.26) | 18(0.26) | 70(0.28) | 38(0.25) | 33(0.31) | 91(0.25) | 88(0.27) | 33(0.27) | 89(0.27) | 37(0.28) | 64(0.3) | 37(0.25) |
| AG | 246(0.51) | 78(0.43) | 123(0.5) | 154(0.54) | 123(0.52) | 36(0.51) | 120(0.48) | 76(0.5) | 45(0.43) | 185(0.52) | 165(0.5) | 57(0.47) | 161(0.48) | 68(0.52) | 104(0.49) | 74(0.49) |
| GG | 118(0.24) | 42(0.23) | 64(0.26) | 70(0.24) | 54(0.23) | 16(0.23) | 59(0.24) | 37(0.25) | 27(0.26) | 83(0.23) | 78(0.24) | 31(0.26) | 85(0.25) | 27(0.2) | 44(0.21) | 40(0.26) |
| G | 482(0.5) | 162(0.44) | 251(0.51) | 294(0.51) | 231(0.49) | 68(0.49) | 238(0.48) | 150(0.5) | 99(0.47) | 351(0.49) | 321(0.48) | 119(0.49) | 331(0.49) | 122(0.46) | 192(0.45) | 154(0.51) |
| rs2239181 | 487 | 184 | 249 | 289 | 238 | 71 | 250 | 152 | 106 | 361 | 332 | 123 | 337 | 133 | 215 | 151 |
| AA | 324(0.67) | 111(0.6) | 164(0.66) | 178(0.62) | 160(0.67) | 37(0.52) | 153(0.61) | 99(0.65) | 61(0.58) | 226(0.63) | 208(0.63) | 72(0.59) | 207(0.61) | 80(0.6) | 127(0.59) | 96(0.64) |
| AC | 141(0.29) | 65(0.35) | 76(0.31) | 99(0.34) | 65(0.27) | 34(0.48) | 85(0.34) | 45(0.3) | 37(0.35) | 123(0.34) | 108(0.33) | 47(0.38) | 117(0.35) | 46(0.35) | 81(0.38) | 48(0.32) |
| CC | 22(0.05) | 8(0.04) | 9(0.04) | 12(0.04) | 13(0.05) | 0 | 12(0.05) | 8(0.05) | 8(0.08) | 12(0.03) | 16(0.05) | 4(0.03) | 13(0.04) | 7(0.05) | 7(0.03) | 7(0.05) |
| C | 185(0.19) | 81(0.22) | 94(0.19) | 123(0.21) | 91(0.19) | 34(0.24) | 109(0.22) | 61(0.2) | 53(0.25) | 147(0.2) | 140(0.21) | 55(0.22) | 143(0.21) | 60(0.23) | 95(0.22) | 62(0.21) |
| rs2107301 | 487 | 187 | 249 | 291 | 238 | 70 | 253 | 155 | 107 | 365 | 337 | 123 | 340 | 135 | 215 | 153 |
| AA | 233(0.48) | 91(0.49) | 115(0.46) | 145(0.5) | 118(0.5) | 37(0.53) | 120(0.47) | 79(0.51) | 56(0.52) | 175(0.48) | 162(0.48) | 66(0.54) | 176(0.52) | 60(0.44) | 105(0.49) | 77(0.5) |
| AG | 215(0.44) | 76(0.41) | 123(0.49) | 117(0.4) | 92(0.39) | 26(0.37) | 105(0.42) | 62(0.4) | 39(0.36) | 153(0.42) | 135(0.4) | 48(0.39) | 130(0.38) | 60(0.44) | 84(0.39) | 60(0.39) |
| GG | 39(0.08) | 20(0.11) | 11(0.04) | 29(0.1) | 28(0.12) | 7(0.1) | 28(0.11) | 14(0.09) | 12(0.11) | 37(0.1) | 40(0.12) | 9(0.07) | 34(0.1) | 15(0.11) | 26(0.12) | 16(0.1) |
| G | 293(0.3) | 116(0.31) | 145(0.29) | 175(0.3) | 148(0.31) | 40(0.29) | 161(0.32) | 90(0.29) | 63(0.29) | 227(0.31) | 215(0.32) | 66(0.27) | 198(0.29) | 90(0.33) | 136(0.32) | 92(0.3) |
| rs1544410 | 487 | 186 | 249 | 291 | 238 | 70 | 253 | 154 | 107 | 364 | 336 | 123 | 339 | 135 | 215 | 153 |
| CC | 445(0.91) | 159(0.85) | 229(0.92) | 263(0.9) | 216(0.91) | 65(0.93) | 221(0.87) | 136(0.88) | 89(0.83) | 327(0.9) | 303(0.9) | 104(0.85) | 296(0.87) | 123(0.91) | 193(0.9) | 131(0.86) |
| CT | 42(0.09) | 27(0.15) | 20(0.08) | 28(0.1) | 22(0.09) | 5(0.07) | 32(0.13) | 18(0.12) | 18(0.17) | 37(0.1) | 33(0.1) | 19(0.15) | 43(0.13) | 12(0.09) | 22(0.1) | 22(0.14) |
| T | 42(0.04) | 27(0.07) | 20(0.04) | 28(0.05) | 22(0.05) | 5(0.04) | 32(0.06) | 18(0.06) | 18(0.08) | 37(0.05) | 33(0.05) | 19(0.08) | 43(0.06) | 12(0.04) | 22(0.05) | 22(0.07) |
| rs757343 | 477 | 180 | 244 | 282 | 233 | 69 | 245 | 148 | 104 | 352 | 323 | 121 | 328 | 131 | 208 | 150 |
| CC | 295(0.62) | 106(0.59) | 141(0.58) | 172(0.61) | 154(0.66) | 40(0.58) | 149(0.61) | 89(0.6) | 59(0.57) | 218(0.62) | 199(0.62) | 70(0.58) | 200(0.61) | 76(0.58) | 124(0.6) | 90(0.6) |
| CT | 159(0.33) | 65(0.36) | 93(0.38) | 96(0.34) | 66(0.28) | 28(0.41) | 84(0.34) | 49(0.33) | 36(0.35) | 120(0.34) | 104(0.32) | 48(0.4) | 113(0.34) | 48(0.37) | 78(0.38) | 50(0.33) |
| TT | 23(0.05) | 9(0.05) | 10(0.04) | 14(0.05) | 13(0.06) | 1(0.01) | 12(0.05) | 10(0.07) | 9(0.09) | 14(0.04) | 20(0.06) | 3(0.02) | 15(0.05) | 7(0.05) | 6(0.03) | 10(0.07) |
| T | 205(0.21) | 83(0.23) | 113(0.23) | 124(0.22) | 92(0.2) | 30(0.22) | 108(0.22) | 69(0.23) | 54(0.26) | 148(0.21) | 144(0.22) | 54(0.22) | 143(0.22) | 62(0.24) | 90(.0.22) | 70(0.23) |
| rs10875692 | 487 | 186 | 249 | 289 | 238 | 70 | 252 | 153 | 106 | 363 | 334 | 124 | 337 | 135 | 216 | 152 |
| CC | 329(0.68) | 135(0.73) | 169(0.68) | 198(0.69) | 160(0.67) | 43(0.61) | 180(0.71) | 110(0.72) | 80(0.75) | 248(0.68) | 229(0.69) | 90(0.73) | 242(0.72) | 88(0.65) | 137(0.63) | 113(0.74) |
| CT | 140(0.29) | 46(0.25) | 71(0.29) | 84(0.29) | 69(0.29) | 24(0.34) | 66(0.26) | 40(0.26) | 23(0.22) | 106(0.29) | 96(0.29) | 31(0.25) | 88(0.26) | 42(0.31) | 72(0.33) | 35(0.23) |
| TT | 18(0.04) | 5(0.03) | 9(0.04) | 7(0.02) | 9(0.04) | 3(0.04) | 6(0.02) | 3(0.02) | 3(0.03) | 9(0.02) | 9(0.03) | 3(0.02) | 7(0.22) | 5(0.04) | 7(0.03) | 4(0.03) |
| T | 176(0.18) | 56(0.15) | 89(0.18) | 98(0.17) | 87(0.18) | 30(0.21) | 78(0.15) | 46(0.15) | 29(0.14) | 124(0.17) | 114(0.17) | 37(0.15) | 102(0.15) | 52(0.19) | 86(0.2) | 43(0.14) |
| rs7975232 | 484 | 177 | 249 | 280 | 235 | 68 | 244 | 145 | 100 | 351 | 319 | 121 | 325 | 129 | 208 | 146 |
| AA | 39(0.08) | 16(0.09) | 21(0.08) | 20(0.07) | 18(0.08) | 2(0.03) | 19(0.08) | 15(0.1) | 15(0.15) | 21(0.06) | 29(0.09) | 7(0.06) | 24(0.07) | 11(0.09) | 11(0.05) | 15(0.1) |
| CA | 177(0.37) | 78(0.44) | 96(0.39) | 114(0.41) | 81(0.34) | 30(0.44) | 105(0.43) | 57(0.39) | 39(0.39) | 148(0.42) | 123(0.39) | 59(0.49) | 137(0.42) | 55(0.43) | 94(0.45) | 61(0.42) |
| CC | 268(0.55) | 83(0.47) | 132(0.53) | 146(0.52) | 136(0.58) | 36(0.53) | 120(0.49) | 73(0.5) | 46(0.46) | 182(0.52) | 167(0.52) | 55(0.45) | 164(0.5) | 63(0.49) | 103(0.5) | 70(0.48) |
| A | 255(0.26) | 110(0.31) | 138(0.28) | 154(0.28) | 117(0.25) | 34(0.25) | 143(0.29) | 87(0.3) | 69(0.34) | 190(0.27) | 181(0.28) | 73(0.3) | 185(0.28) | 77(0.3) | 116(0.28) | 91(0.31) |
| rs731236 | 487 | 186 | 249 | 289 | 238 | 70 | 252 | 153 | 106 | 363 | 334 | 124 | 337 | 135 | 216 | 152 |
| AA | 442(0.91) | 160(0.86) | 226(0.91) | 262(0.91) | 216(0.91) | 64(0.91) | 224(0.89) | 134(0.88) | 91(0.86) | 325(0.9) | 302(0.9) | 105(0.85) | 298(0.88) | 121(0.9) | 194(0.9) | 131(0.86) |
| AG | 44(0.09) | 24(0.13) | 23(0.09) | 27(0.09) | 21(0.09) | 6(0.09) | 27(0.11) | 18(0.12) | 14(0.13) | 37(0.1) | 31(0.09) | 18(0.15) | 37(0.11) | 14(0.1) | 20(0.09) | 21(0.14) |
| GG | 1(0) | 2(0.01) | 0 | 0 | 1(0) | 0 | 1(0) | 1(0.01) | 1(0.01) | 1(0) | 1(0) | 1(0.01) | 2(0.01) | 0 | 2（0.01） | 0 |
| G | 46(0.05) | 28(0.08) | 23(0.05) | 27(0.05) | 23(0.05) | 6(0.04) | 29(0.06) | 20(0.07) | 16(0.08) | 39(0.05) | 33(0.05) | 20(0.08) | 41(0.06) | 14(0.05) | 24(0.06) | 21(0.07) |
| rs739837 | 479 | 175 | 244 | 275 | 235 | 67 | 238 | 145 | 101 | 344 | 313 | 119 | 318 | 129 | 200 | 145 |
| GG | 265(0.55) | 84(0.48) | 129(0.53) | 142(0.52) | 136(0.58) | 35(0.52) | 118(0.5) | 73(0.5) | 46(0.46) | 179(0.52) | 164(0.52) | 55(0.46) | 160(0.5) | 64(0.5) | 100(0.5) | 69(0.48) |
| GT | 174(0.36) | 75(0.43) | 94(0.39) | 113(0.41) | 80(0.34) | 30(0.45) | 101(0.42) | 57(0.39) | 40(0.4) | 144(0.42) | 120(0.38) | 57(0.48) | 134(0.42) | 54(0.42) | 89(0.44) | 61(0.42) |
| TT | 40(0.08) | 16(0.09) | 21(0.09) | 20(0.07) | 19(0.08) | 2(0.03) | 19(0.08) | 15(0.1) | 15(0.15) | 21(0.06) | 29(0.09) | 7(0.06) | 24(0.08) | 11(0.09) | 11(0.06) | 15(0.1) |
| T | 254(0.27) | 107(0.31) | 136(0.28) | 153(0.28) | 118(0.25) | 34(0.25) | 139(0.29) | 87(0.3) | 70(0.35) | 186(0.27) | 178(0.28) | 71(0.3) | 182(0.29) | 76(0.29) | 111(0.28) | 91(0.31) |
| rs3847987 | 487 | 186 | 249 | 289 | 238 | 70 | 252 | 153 | 106 | 363 | 334 | 124 | 337 | 135 | 216 | 152 |
| AA | 24(0.05) | 9(0.05) | 10(0.04) | 15(0.05) | 14(0.06) | 1(0.01) | 13(0.05) | 10(0.07) | 10(0.09) | 14(0.04) | 21(0.06) | 3(0.02) | 15(0.04) | 8(0.06) | 7(0.03) | 10(0.07) |
| CA | 163(0.33) | 69(0.37) | 95(0.38) | 101(0.35) | 68(0.29) | 27(0.39) | 90(0.36) | 53(0.35) | 37(0.35) | 128(0.35) | 111(0.33) | 51(0.41) | 119(0.35) | 51(0.38) | 83(0.38) | 53(0.35) |
| CC | 300(0.62) | 108(0.58) | 144(0.58) | 173(0.6) | 156(0.66) | 42(0.6) | 149(0.59) | 90(0.59) | 59(0.56) | 221(0.61) | 202(0.6) | 70(0.56) | 203(0.6) | 76(0.56) | 126(0.58) | 89(0.59) |
| A | 211(0.22) | 87(0.23) | 115(0.23) | 131(0.23) | 96(0.2) | 29(0.21) | 116(0.23) | 73(0.24) | 57(0.27) | 156(0.21) | 153(0.23) | 57(0.23) | 149(0.22) | 67(0.25) | 97(0.22) | 73(0.24) |
| rs9729 | 485 | 186 | 247 | 291 | 238 | 71 | 253 | 153 | 107 | 364 | 336 | 123 | 340 | 134 | 216 | 153 |
| GG | 256(0.53) | 91(0.49) | 128(0.52) | 144(0.49) | 128(0.54) | 33(0.46) | 124(0.49) | 78(0.51) | 48(0.45) | 186(0.51) | 168(0.5) | 59(0.48) | 169(0.5) | 64(0.48) | 107(0.5) | 72(0.47) |
| GT | 192(0.4) | 75(0.4) | 99(0.4) | 121(0.42) | 93(0.39) | 35(0.49) | 106(0.42) | 55(0.36) | 41(0.38) | 150(0.41) | 131(0.39) | 55(0.45) | 139(0.41) | 57(0.43) | 93(0.43) | 63(0.41) |
| TT | 37(0.08) | 20(0.11) | 20(0.08) | 26(0.09) | 17(0.07) | 3(0.04) | 23(0.09) | 20(0.13) | 18(0.17) | 28(0.08) | 37(0.11) | 9(0.07) | 32(0.09) | 13(0.1) | 16(0.07) | 18(0.12) |
| T | 266(0.27) | 115(0.31) | 139(0.28) | 173(0.3) | 127(0.27) | 41(0.29) | 152(0.3) | 95(0.31) | 77(0.36) | 206(0.28) | 205(0.31) | 73(0.3) | 203(0.3) | 83(0.31) | 125(0.29) | 99(0.32) |

Table S2 | Frequencies of alleles and genotypes in MG subgroups and the control group (comprehensive classification)

|  | Control | Juvenile | Adult |  |  |  |  |  |  |  |  |  |  |
| --- | --- | --- | --- | --- | --- | --- | --- | --- | --- | --- | --- | --- | --- |
|  |  |  |  | Thymoma(+) | Thymoma(-) | | | | | | | | |
|  |  |  |  |  |  | AChRAb(-) | | | AChRAb(+) | | | | |
|  |  |  |  |  |  | total | Musk(+) | Musk(-) | total | 15-50 | ＞50 | ocular | Generalized |
| Number | 487 | 71 | 409 | 104 | 300 | 84 | 15 | 69 | 204 | 126 | 78 | 135 | 67 |
| rs4516035 | 484 | 71 | 404 | 103 | 296 | 83 | 15 | 68 | 201 | 126 | 75 | 132 | 67 |
| TT | 456(0.94) | 67(0.94) | 387(0.96) | 97(0.94) | 285(0.96) | 80(0.96) | 15(1) | 65(0.96) | 193(0.96) | 122(0.97) | 71(0.95) | 127(0.96) | 64(0.96) |
| TC | 27(0.06) | 4(0.06) | 17(0.04) | 6(0.06) | 11(0.04) | 3(0.04) | 0(0) | 3(0.04) | 8(0.04) | 4(0.03) | 4(0.05) | 5(0.04) | 3(0.04) |
| CC | 1(0) |  |  |  |  |  |  |  |  |  |  |  |  |
| C | 29(0.03) | 4(0.03) | 17(0.02) | 6(0.03) | 11(0.02) | 3(0.02) | 0(0) | 3(0.02) | 8(0.02) | 4(0.02) | 4(0.03) | 5(0.02) | 3(0.02) |
| rs7136534 | 486 | 69 | 405 | 103 | 297 | 84 | 15 | 69 | 201 | 126 | 75 | 132 | 67 |
| CC | 184(0.38) | 22(0.32) | 156(0.39) | 40(0.39) | 115(0.39) | 35(0.42) | 5(0.33) | 30(0.43) | 74(0.37) | 49(0.39) | 25(0.33) | 48(0.36) | 24(0.36) |
| CT | 233(0.48) | 36(0.52) | 182(0.45) | 45(0.44) | 135(0.45) | 35(0.42) | 4(0.27) | 31(0.45) | 95(0.47) | 58(0.46) | 37(0.49) | 62(0.47) | 33(0.49) |
| TT | 69(0.114) | 11(0.16) | 67(0.17) | 18(0.17) | 47(0.16) | 14(0.17) | 6(0.4) | 8(0.12) | 32(0.16) | 19(0.15) | 13(0.17) | 22(0.17) | 10(0.15) |
| T | 371(0.38) | 58(0.42) | 316(0.39) | 81(0.39) | 229(0.39) | 63(0.38) | 16(0.53) | 47(0.34) | 159(0.4) | 96(0.38) | 63(0.42) | 106(0.4) | 53(0.4) |
| rs11574027 | 486 | 71 | 404 | 103 | 296 | 83 | 15 | 68 | 201 | 126 | 75 | 132 | 67 |
| AA | 9(0.02) | 0(0) | 4(0.01) | 2(0.02) | 2(0.01) | 1(0.01) | 0(0) | 1(0.01) | 1(0) | 1(0.01) | 0(0) | 1(0.01) | 0(0) |
| CA | 102(0.21) | 13(0.18) | 100(0.25) | 22(0.21) | 76(0.26) | 23(0.28) | 2(0.13) | 21(0.31) | 52(0.26) | 33(0.26) | 19(0.25) | 32(0.24) | 19(0.28) |
| CC | 375(0.77) | 58(0.82) | 300(0.74) | 79(0.77) | 218(0.74) | 59(0.71) | 13(0.87) | 46(0.68) | 148(0.74) | 92(0.73) | 56(0.75) | 99(0.75) | 48(0.72) |
| A | 120(0.12) | 13(0.09) | 108(0.13) | 26(0.13) | 80(0.14) | 25(0.15) | 2(0.07) | 23(0.17) | 54(0.13) | 35(0.14) | 19(0.13) | 34(0.13) | 19(0.14) |
| rs2238136 | 487 | 71 | 404 | 103 | 296 | 83 | 16 | 68 | 201 | 126 | 75 | 132 | 67 |
| CC | 314(0.64) | 47(0.66) | 257(0.64) | 63(0.61) | 190(0.64) | 48(0.58) | 12(0.8) | 36(0.53) | 133(0.66) | 83(0.66) | 50(0.67) | 89(0.67) | 42(0.63) |
| CT | 147(0.3) | 21(0.3) | 134(0.33) | 34(0.33) | 99(0.33) | 33(0.4) | 3(0.2) | 30(0.44) | 63(0.31) | 41(0.33) | 22(0.29) | 40(0.3) | 23(0.34) |
| TT | 26(0.05) | 3(0.04) | 13(0.03) | 6(0.06) | 7(0.02) | 2(0.02) | 0(0) | 2(0.03) | 5(0.02) | 2(0.02) | 3(0.04) | 3(0.02) | 2(0.03) |
| T | 199(0.2) | 27(0.19) | 160(0.2) | 46(0.22) | 113(0.19) | 37(0.22) | 3(0.1) | 34(0.25) | 73(0.18) | 45(0.18) | 28(0.19) | 46(0.17) | 27(0.2) |
| rs2228570 | 486 | 71 | 401 | 102 | 294 | 83 | 15 | 68 | 199 | 124 | 75 | 130 | 67 |
| AA | 102(0.21) | 12(0.17) | 67(0.17) | 19(0.19) | 48(0.16) | 11(0.13) | 0(0) | 11(0.16) | 33(0.17) | 20(0.16) | 13(0.17) | 22(0.17) | 10(0.15) |
| GA | 239(0.49) | 35(0.49) | 220(0.55) | 56(0.55) | 161(0.55) | 53(0.64) | 14(0.93) | 39(0.57) | 103(0.52) | 62(0.5) | 41(0.55) | 72(0.55) | 30(0.45) |
| GG | 145(0.3) | 24(0.34) | 114(0.28) | 27(0.26) | 85(0.29) | 19(0.23) | 1(0.07) | 18(0.26) | 63(0.32) | 42(0.34) | 21(0.28) | 36(0.28) | 27(0.4) |
| A | 443(0.46) | 59(0.42) | 354(0.44) | 94(0.46) | 257(0.44) | 75(0.45) | 14(0.47) | 61(0.45) | 169(0.42) | 102(0.41) | 67(0.45) | 116(0.45) | 50(0.37) |
| rs2239186 | 486 | 70 | 400 | 102 | 293 | 82 | 15 | 67 | 199 | 124 | 75 | 131 | 66 |
| AA | 122(0.25) | 18(0.26) | 108(0.27) | 31(0.3) | 75(0.26) | 21(0.26) | 3(0.2) | 18(0.27) | 50(0.25) | 32(0.26) | 18(0.24) | 37(0.28) | 13(0.2) |
| AG | 246(0.51) | 36(0.51) | 196(0.49) | 44(0.43) | 151(0.52) | 39(0.48) | 8(0.53) | 31(0.46) | 107(0.54) | 64(0.52) | 43(0.57) | 67(0.51) | 38(0.58) |
| GG | 118(0.24) | 16(0.23) | 96(0.24) | 27(0.26) | 67(0.23) | 22(0.27) | 4(0.27) | 18(0.27) | 42(0.21) | 28(0.23) | 14(0.19) | 27(0.21) | 15(0.23) |
| G | 482(0.5) | 68(0.49) | 388(0.48) | 98(0.48) | 285(0.49) | 83(0.51) | 16(0.47) | 67(0.5) | 191(0.48) | 120(0.48) | 71(0.47) | 121(0.46) | 68(0.52) |
| rs2239181 | 487 | 71 | 402 | 103 | 294 | 83 | 15 | 68 | 199 | 124 | 75 | 131 | 66 |
| AA | 324(0.67) | 37(0.52) | 252(0.63) | 59(0.57) | 191(0.65) | 52(0.63) | 7(0.47) | 45(0.66) | 131(0.66) | 79(0.64) | 52(0.69) | 82(0.63) | 48(0.73) |
| AC | 141(0.29) | 34(0.48) | 130(0.32) | 36(0.35) | 91(0.31) | 30(0.36) | 7(0.47) | 23(0.34) | 57(0.29) | 39(0.31) | 18(0.24) | 41(0.31) | 15(0.23) |
| CC | 22(0.05) | 0(0) | 20(0.05) | 8(0.08) | 12(0.04) | 1(0.01) | 1(0.07) | 0(0) | 11(0.06) | 6(0.05) | 5(0.07) | 8(0.06) | 3(0.05) |
| C | 185(0.19) | 34(0.24) | 170(0.21) | 52(0.25) | 115(0.2) | 32(0.19) | 9(0.3) | 23(0.17) | 79(0.2) | 51(0.21) | 28(0.19) | 57(0.22) | 21(0.16) |
| rs2107301 | 487 | 70 | 408 | 104 | 299 | 84 | 15 | 69 | 203 | 126 | 77 | 134 | 67 |
| AA | 233(0.48) | 37(0.53) | 199(0.49) | 56(0.54) | 139(0.46) | 42(0.5) | 8(0.53) | 34(0.49) | 93(0.46) | 56(0.44) | 37(0.48) | 64(0.48) | 29(0.43) |
| AG | 215(0.44) | 26(0.37) | 167(0.41) | 37(0.36) | 129(0.43) | 36(0.43) | 6(0.4) | 30(0.43) | 85(0.42) | 54(0.43) | 31(0.4) | 55(0.41) | 28(0.42) |
| GG | 39(0.08) | 7(0.1) | 42(0.1) | 11(0.11) | 31(0.1) | 6(0.07) | 1(0.07) | 5(0.07) | 25(0.12) | 16(0.13) | 9(0.12) | 15(0.11) | 10(0.15) |
| G | 293(0.3) | 40(0.29) | 251(0.31) | 59(0.28) | 191(0.32) | 48(0.29) | 8(0.27) | 40(0.29) | 135(0.33) | 86(0.34) | 49(0.32) | 85(0.32) | 48(0.36) |
| rs1544410 | 487 | 70 | 407 | 104 | 298 | 84 | 15 | 69 | 202 | 126 | 76 | 133 | 67 |
| CC | 445(0.91) | 65(0.93) | 357(0.88) | 88(0.85) | 264(0.89) | 68(0.81) | 12(0.8) | 56(0.81) | 187(0.93) | 117(0.93) | 70(0.92) | 121(0.91) | 64(0.96) |
| CT | 42(0.39) | 5(0.07) | 50(0.12) | 16(0.15) | 34(0.11) | 16(0.19) | 3(0.2) | 13(0.19) | 15(0.07) | 9(0.07) | 6(0.04) | 12(0.09) | 3(0.04) |
| T | 42(0.04) | 5(0.04) | 50(0.06) | 16(0.08) | 34(0.06) | 16(0.1) | 3(0.1) | 13(0.09) | 15(0.04) | 9(0.04) | 6(0.04) | 12(0.05) | 3(0.02) |
| rs757343 | 477 | 69 | 393 | 101 | 287 | 81 | 15 | 66 | 194 | 121 | 73 | 125 | 67 |
| CC | 295(0.62) | 40(0.58) | 238(0.61) | 57(0.56) | 180(0.63) | 51(0.63) | 7(0.47) | 44(0.67) | 121(0.62) | 74(0.61) | 47(0.64) | 76(0.61) | 44(0.66) |
| CT | 159(0.33) | 28(0.41) | 133(0.34) | 35(0.35) | 94(0.33) | 29(0.36) | 7(0.47) | 22(0.33) | 61(0.31) | 40(0.33) | 21(0.29) | 41(0.33) | 20(0.3) |
| TT | 23(0.05) | 1(0.01) | 22(0.06) | 9(0.09) | 13(0.05) | 1(0.01) | 1(0.07) | 0(0) | 12(0.06) | 7(0.06) | 5(0.07) | 8(0.06) | 3(0.04) |
| T | 205(0.21) | 30(0.22) | 177(0.23) | 53(0.26) | 120(0.21) | 31(0.19) | 9(0.3) | 22(0.17) | 85(0.22) | 54(0.22) | 31(0.21) | 57(0.23) | 26(0.19) |
| rs10875692 | 487 | 70 | 405 | 103 | 297 | 84 | 15 | 69 | 201 | 126 | 75 | 132 | 67 |
| CC | 329(0.68) | 43(0.61) | 290(0.72) | 77(0.75) | 209(0.7) | 63(0.75) | 12(0.8) | 51(0.74) | 137(0.68) | 88(0.7) | 49(0.65) | 94(0.71) | 41(0.61) |
| CT | 140(0.29) | 24(0.34) | 106(0.26) | 23(0.22) | 82(0.28) | 19(0.23) | 2(0.13) | 17(0.25) | 60(0.3) | 36(0.29) | 24(0.32) | 37(0.28) | 23(0.34) |
| TT | 18(0.04) | 3(0.04) | 9(0.02) | 3(0.03) | 6(0.02) | 2(0.02) | 1(0.07) | 1(0.01) | 4(0.02) | 2(0.02) | 2(0.03) | 1(0.01) | 3(0.04) |
| T | 176(0.18) | 30(0.21) | 124(0.15) | 29(0.14) | 94(0.16) | 23(0.14) | 4(0.13) | 19(0.14) | 68(0.17) | 40(0.16) | 28(0.19) | 39(0.15) | 29(0.22) |
| rs7975232 | 484 | 68 | 389 | 97 | 287 | 82 | 15 | 67 | 193 | 122 | 71 | 125 | 66 |
| AA | 39(0.08) | 2(0.03) | 34(0.09) | 14(0.14) | 20(0.07) | 4(0.05) | 2(0.13) | 2(0.03) | 16(0.08) | 9(0.07) | 7(0.1) | 12(0.1) | 3(0.05) |
| CA | 177(0.37) | 30(0.44) | 162(0.42) | 38(0.39) | 120(0.42) | 41(0.5) | 7(0.47) | 34(0.51) | 73(0.38) | 47(0.39) | 26(0.37) | 47(0.38) | 26(0.39) |
| CC | 268(0.55) | 36(0.53) | 193(0.5) | 45(0.46) | 147(0.51) | 37(0.45) | 6(0.4) | 31(0.46) | 104(0.54) | 66(0.54) | 38(0.54) | 66(0.53) | 37(0.56) |
| A | 255(0.26) | 34(0.25) | 230(0.3) | 66(0.34) | 160(0.28) | 49(0.3) | 11(0.37) | 38(0.28) | 105(0.27) | 65(0.27) | 40(0.28) | 71(0.28) | 32(0.24) |
| rs731236 | 487^a^ | 70 | 405 | 103 | 297 | 84^ab^ | 15 | 69 | 201^b^ | 126 | 75 | 132 | 67 |
| AA | 442(0.91) | 64(0.91) | 358(0.88) | 90(0.87) | 263(0.89) | 67(0.8) | 13(0.87) | 54(0.78) | 185(0.92) | 120(0.95) | 65(0.87) | 121(0.92) | 62(0.93) |
| AG | 44(0.09) | 6(0.09) | 45(0.11) | 12(0.12) | 33(0.11) | 16(0.19) | 2(0.13) | 14(0.2) | 16(0.08) | 6(0.05) | 10(0.13) | 11(0.08) | 5(0.07) |
| GG | 1(0) | 0(0) | 2(0) | 1(0.01) | 1(0) | 1(0.01) | 0(0) | 1(0.01) | 0(0) |  |  |  |  |
| G | 46(0.05) | 6(0.04) | 49(0.06) | 14(0.07) | 35(0.06) | 18(0.11) | 2(0.07) | 16(0.12) | 16(0.04) | 6(0.02) | 10(0.07) | 11(0.04) | 5(0.04) |
| rs739837 | 479 | 67 | 383 | 98 | 281 | 80 | 15 | 65 | 189 | 117 | 72 | 121 | 66 |
| GG | 265(0.55) | 35(0.52) | 191(0.5) | 45(0.46) | 145(0.52) | 37(0.46) | 6(0.4) | 31(0.48) | 102(0.54) | 64(0.55) | 38(0.53) | 63(0.52) | 38(0.58) |
| GT | 174(0.36) | 30(0.45) | 158(0.41) | 39(0.4) | 116(0.41) | 39(0.49) | 7(0.47) | 32(0.49) | 71(0.38) | 44(0.38) | 27(0.38) | 46(0.38) | 25(0.38) |
| TT | 40(0.08) | 2(0.03) | 34(0.09) | 14(0.14) | 20(0.07) | 4(0.05) | 2(0.13) | 2(0.03) | 16(0.08) | 9(0.08) | 7(0.1) | 12(0.1) | 3(0.05) |
| T | 254(0.27) | 34(0.25) | 226(0.3) | 67(0.34) | 156(0.28) | 47(0.29) | 11(0.37) | 36(0.28) | 103(0.27) | 62(0.26) | 41(0.28) | 70(0.29) | 31(0.23) |
| rs3847987 | 487 | 70 | 405 | 103 | 297 | 84 | 15 | 69 | 201 | 126 | 75 | 132 | 67 |
| AA | 24(0.05) | 1(0.01) | 23(0.06) | 10(0.1) | 13(0.04) | 1(0.01) | 1(0.07) | 0(0) | 12(0.06) | 7(0.06) | 5(0.07) | 8(0.06) | 3(0.04) |
| CA | 163(0.33) | 27(0.39) | 143(0.35) | 36(0.35) | 103(0.35) | 32(0.38) | 7(0.47) | 25(0.36) | 67(0.33) | 45(0.36) | 22(0.29) | 46(0.35) | 21(0.31) |
| CC | 300(0.62) | 42(0.6) | 239(0.59) | 57(0.55) | 181(0.61) | 51(0.61) | 7(0.47) | 44(0.64) | 122(0.61) | 74(0.59) | 48(0.64) | 78(0.59) | 43(0.64) |
| A | 211(0.22) | 29(0.21) | 189(0.23) | 56(0.27) | 129(0.22) | 34(0.2) | 9(0.3) | 25(0.18) | 91(0.23) | 59(0.23) | 32(0.21) | 62(0.23) | 27(0.2) |
| rs9729 | 485 | 71 | 406 | 104 | 297 | 83 | 15 | 68 | 202 | 126 | 76 | 133 | 67 |
| GG | 256(0.53) | 33(0.46) | 202(0.5) | 47(0.45) | 154(0.52) | 41(0.49) | 6(0.4) | 35(0.51) | 106(0.52) | 67(0.53) | 39(0.51) | 67(0.5) | 38(0.57) |
| GT | 192(0.4) | 35(0.49) | 161(0.4) | 40(0.38) | 117(0.39) | 36(0.43) | 7(0.47) | 29(0.43) | 76(0.38) | 48(0.38) | 28(0.37) | 51(0.38) | 25(0.37) |
| TT | 37(0.08) | 3(0.04) | 43(0.11) | 17(0.16) | 26(0.09) | 6(0.07) | 2(0.13) | 4(0.06) | 20(0.1) | 11(0.09) | 9(0.12) | 15(0.11) | 4(0.06) |
| T | 266(0.27) | 41(0.29) | 247(0.3) | 74(0.36) | 169(0.28) | 48(0.29) | 11(0.37) | 37(0.27) | 116(0.29) | 70(0.28) | 46(0.3) | 81(0.3) | 33(0.25) |

a Adult thymoma (-) AChRAb (-) MG vs control group, p=0.002, OR=2.42(1.37-3.75), P_bon=_0.032

b Adult thymoma (-) AChRAb (-) MG vs adult thymoma (-) AChRAb (+) MG, p=0.002, OR=2.90(1.44-4.82), P_bon=_0.032
